# The Single Intradermal Cervical Comparative Test Interferes with Johne’s Disease ELISA Diagnostics

**DOI:** 10.3389/fimmu.2014.00564

**Published:** 2014-11-12

**Authors:** Aideen E. Kennedy, Ana T. Da Silva, Noel Byrne, Rodney Govender, John MacSharry, Jim O’Mahony, Riona G. Sayers

**Affiliations:** ^1^Animal and Bioscience Research Department, Animal and Grassland Research and Innovation Centre, Teagasc, Cork, Ireland; ^2^Department of Biological Sciences, Cork Institute of Technology, Cork, Ireland; ^3^Alimentary Pharmabiotic Centre, Biosciences Institute, University College Cork, Cork, Ireland

**Keywords:** Mycobacteriacea, Johne’s disease, TB test, ELISA, PPD

## Abstract

Enzyme-linked immunosorbent assays (ELISA) of milk and serum samples are a routinely used method of screening herds for *Mycobacterium avium* subspecies *paratuberculosis* (MAP). Infection with MAP causes granulomatous enteritis of ruminants known as Johne’s disease (JD). The sensitivity (Se) and specificity (Sp) of MAP ELISAs leads to difficulties in the identification of both infected and infectious animals. Interference with MAP ELISA Se and Sp has been reported in MAP seronegative cows following administration of purified protein derivative (PPD) as part of intradermal testing for bovine tuberculosis (bTB). The aim of this study is to examine the impact of the single intradermal cervical comparative test (SICCT) for bTB, on both serum and milk MAP ELISA tests, in a herd containing both seropositive and seronegative cows pre-SICCT. A secondary objective is to provide appropriate timing of JD ELISA tests in relation to the SICCT. A herd of 139 cows were serum and milk sampled pre- and post-SICCT administration. Prior to SICCT, 6% of the herd tested seropositive for MAP using milk ELISA, with 8% positive on serum. ID Screen Paratuberculosis Indirect Screening Test (ID Vet) was used to screen the herd. Within 14 days of PPD administration, a significant increase in the prevalence of seropositive cows was recorded. Identical prevalence’s were recorded with both test matrices (39%). ELISA values remained significantly higher until day 43 post-SICCT in milk (*P* = 0.850), and day 71 in serum (*P* = 0.602). If the “new” positives detected post-bTB testing are deemed false positives due to generation of cross-reacting antibodies by administration of PPD, milk would appear a more suitable sample for JD ELISA testing within 2 months of SICCT. In summary, sampling for JD utilizing milk ELISA should be avoided in the 43-day period following PPD administration, with serum ELISA sampling avoided for an additional 28 days.

## Introduction

*Mycobacterium avium* subspecies *paratuberculosis* (MAP), a member of the Mycobacteriacea family, causes chronic granulomatous enteritis known as Johne’s disease (JD) (1). Clinical JD is characterized by diarrhea and progressive cachexia, which ultimately results in death (2). Uncertainty exists regarding a potential causal link between MAP and Crohn’s disease in humans (3, 4). The potential damage to the global dairy industry, should a link between Crohn’s and MAP be fully substantiated (5), combined with impacts on animal health, has prompted the establishment of JD control programs in a number of countries (6–8).

Use of enzyme-linked immunosorbent assays (ELISA) to identify animals at risk of being infected with MAP is common in control programs internationally (8, 9), including Ireland (10). ELISA is favored as a screening test due to its relatively low cost compared to fecal culture or polymerase chain reaction (PCR) (11). ELISAs also provide timely results compared to culture methods (11). The sensitivity (Se) and specificity (Sp) of MAP ELISAs, however, leads to difficulties in the identification of both infected and infectious individuals (12).

*Mycobacterium bovis*, the causative agent of bovine tuberculosis (bTB), is an additional pathogenic and definitively zoonotic (13) member of the Mycobacteriaceae. To reduce the zoonotic risk posed by bTB, address public/animal health concerns, and limit trade restrictions, a compulsory national eradication program for bTB was established in Ireland in 1962 (14). This eradication program involves ante-mortem testing of all registered bovines annually using the single intradermal cervical comparative test (SICCT) and post-mortem carcass inspection. All SICCT positive animals (reactors) are slaughtered, the herd of origin is restricted, and additional bTB testing is applied to the herd. The comprehensive nature of the testing program can lead to some animals being tested up to five times in a single year (15).

The SICCT utilizes intradermal introduction of *M. bovis* and *M. avium* subsp. *avium* purified protein derivatives (bPPD and aPPD) at two different sites on the neck to elicit a delayed hypersensitivity response mediated by T cells (16). Comparative measurements at both injection sites, taken 72 h post-PPD administration, are used to assess infection status (16). Additional ante-mortem testing methods used internationally for detection of bTB include the single intradermal test and the caudal fold test, both less specific than SICCT (17).

Members of the Mycobacteriaceae family share several antigens, which can lead to diagnostic difficulties due to antibody cross reaction (18). MAP infection can interfere with specificity of bTB diagnostics (19), and likewise *M. bovis* infection can affect MAP serological tests (20). Varges et al. (21), has also shown interference by both single and comparative intradermal bTB tests on MAP sero diagnostics in bTB negative animals. The primary purpose of this current study was to investigate the impact of SICCT on the prevalence of ELISA positive results (serum and milk) in an Irish herd containing both MAP ELISA seropositive and seronegative animals over a period of 6 months. Secondary objectives included comparing milk and serum ELISA readings and investigating whether serum samples could be taken at the 72 h bTB visit without interference from PPD administration.

## Materials and Methods

### Study herd

A 139-cow spring-calving dairy herd (mean-calving date February 19th) was recruited. This herd was depopulated in 1997 following a confirmed case of bovine spongiform encephalopathy (BSE). The experimental herd, therefore, consisted of descendants of cows used to repopulate the farm in 1998 (22). Annual statutory bTB test results were sourced from 1998 to provide a bTB history for the herd. Veterinary records were obtained in order to record a JD history for the herd post-repopulation. Approximately 60% of the cows were Holstein Friesian (HF), the remaining 40% purebred Jersey (Je) or Je cross-breeds. The study was licensed by the Irish Department of Health and Children.

### Sample collection

Milk and serum samples were collected 10 and 13 days prior to administration of the compulsory annual SICCT herd test in May 2012 (pre-SICCT). The SICCT was administered by the Department of Agriculture, Food and the Marine (DAFM) approved private veterinary practitioner (PVP) responsible for the care of animals on this farm as is standard practice for the Irish national bTB eradication scheme. Milk and serum samples were collected every 14 days (approximately) for 2 months post-SICCT and on a monthly basis thereafter until the composition of the herd changed materially due to end of lactation culling (longitudinal data). Sampling dates for serum and milk samples are outlined in Table 1. A limit of a 7-day interval between serum and milk sampling was applied in order to consider samples as “matched.” Milk samples were not available for all cows at every sampling time point which is reflected as small variations in sample sizes. Additionally, milk samples were not collected in September 2012 due to an un-related health issue on farm. Fecal samples were collected on a weekly basis from consistently ELISA positive cows from 90 days post-SICCT. These cows were also subjected to a veterinary clinical exam.

**Table 1:** **Timetable of serum and milk samples and dates of SICCT**.

	Serum sampling date	Milk sampling date	Days post-PPD administration
Pre SICCT	May 29	May 31	
SICCT test day 1 PPD administration	June 11		0
SICCT day 2	June 14		3
SICCT day 2 – serum sample only	June 14		3
Post SICCT Match 1		June 20	9
	June 25		14
Post SICCT Match 2	July 11	July 11	30
Post SICCT Match 3	July 24	July 24	43
Post SICCT Match 4	August 8	August 8	58
Post SICCT Match 5	August 21	August 21	71
	September 5	No sample	99
Post SICCT Match 6	October 1	October 1	112
Post SICCT Match 7	November 1	November 1	143

Serum and milk samples were tested using a commercial ISO17025 accredited laboratory (designated laboratory for Irish voluntary JD control program) using the ID Screen Paratuberculosis Indirect Screening Test (ID Vet, Montpellier, France). The test is an *M. phlei* absorbed ELISA detecting anti-MAP IgG. Status of the sample was evaluated by examining the sample to positive ratio (*S*/*P* ratio) calculated using the formula *S*/*P* Ratio = [(OD_Sample_ − OD_Positive control_) ÷ (OD_Positive control_ − OD_Negative control_) × 100]. Fecal samples were tested by microbial culture and real-time PCR (rtRT-PCR) using “in-house” methodologies developed by Cork Institute of Technology as outlined by Douarre et al. (23). The target gene was IS900. Primer sequences for the amplification were 5’-GAAGGGTGTTCGGGGCCGTCGCTTAGG-3’ and 5’-GGCGTTGAGGTCGATCGCCC ACGTGAC-3’ (reverse primer).

### Data analysis

Descriptive analysis, dataset construction, and graphical representations were completed in Excel (MS Office 2010). Normality of datasets was examined visually using ladders of power histograms in Stata (version 12). Additional statistical analyses including chi-squared test, *t*-test, box plot construction, Spearman rank correlation, and generalized estimating equations (GEE) were completed using Stata (version 12).

For the purposes of reporting within-herd MAP prevalence, ELISA *S*/*P* ratio results were interpreted according to manufacture instructions, i.e., ≥70 *S*/*P* (serum) and ≥15 *S*/*P* (milk) were categorized as positive, with a single exception. Cows recording *S*/*P* ratios of 60 ≥ SP < 70 (normally classified as inconclusive), were also categorized as negative. The prevalence of positive cows within the herd was plotted vs. trial day. Box plots were constructed to highlight trends in ELISA *S*/*P* % readings pre- and post-SICCT

Longitudinal milk and serum ELISA results were used to create datasets for statistical analysis. ELISA results were recorded as both a categorical variable (positive, negative) and a continuous variable (ELISA *S*/*P* readings). Multivariable GEE was used to investigate differences between pre- and post-SICCT categorical and continuous variables (dependent variables). Independent variables included in the models were sampling time point (pre-SICCT, post-SICCT), breed (Friesian, Jersey), parity (parities 1, 2, 3, ≥4), and date of calving (January, February, March, April). Second level interactions between independent variables were examined and included in the model at *P* ≤ 0.05. For categorical variable analysis, a binomial distribution was assumed and a logit link function used. For continuous variable analysis, a Gaussian distribution and an identity link function was used. An exchangeable correlation was applied to both analyses. To investigate the correlation between milk and serum ELISA results, Spearman correlation (*r_s_*) was performed on categorical data sets.

## Results

Results of statutory bTB testing for this farm over the past 8 years indicate minimal issues with bTB in this herd. Similarly, no bTB positive reactor was identified following SICCT in 2012. From herd repopulation in 1998 to commencement of this study, no clinical case of JD had been diagnosed on the study farm.

**Figure 1:**
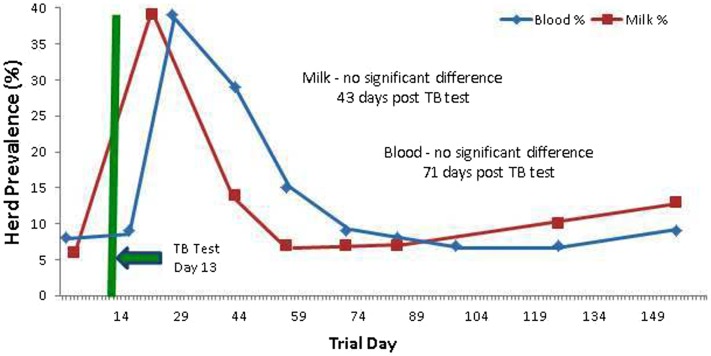
**Percentage (%) of the herd testing positive on Johne’s disease ELISAs (milk and serum) at different trial days, both pre and post the administration of the TB test**. An increased number of positives are identified post TB test administration.

**Figure 2:**
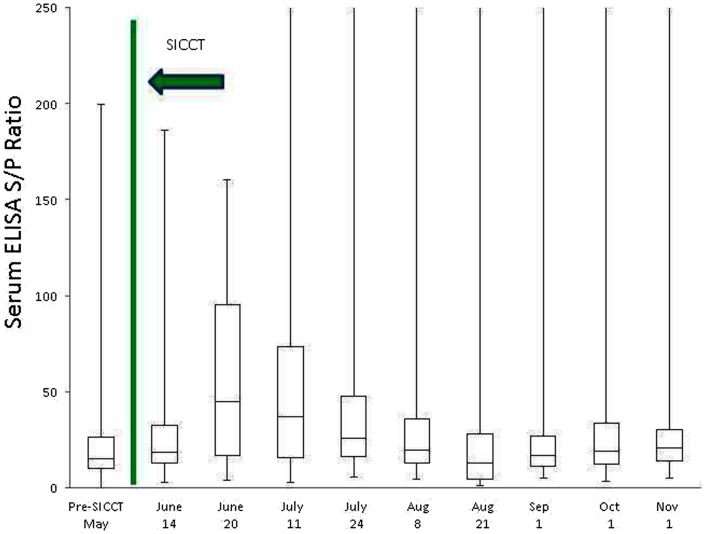
**Box plot identifying differences in serum ELISA *S*/*P* ratios at different sampling points, both pre and post the administration of the TB test**. To improve visualization of interquartile ranges, only *S*/*P* values <250 are shown.

**Figure 3:**
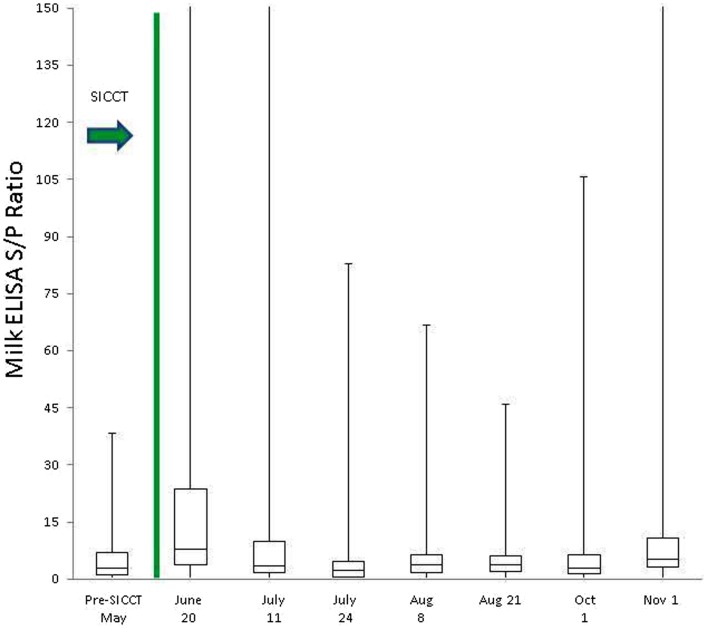
**Box plot identifying differences in milk ELISA *S*/*P* ratios at different sampling points, both pre and post the administration of the TB test**. To improve visualization of interquartile ranges, only *S*/*P* values <150 shown.

Prior to administration of the SICCT, a total of 11 of 139 cows (7.9%) tested MAP ELISA positive in serum, with 8 of 137 (5.8%) milk samples testing positive. Following administration of SICCT, a significant increase in the prevalence of ELISA positives was recorded on both test matrices (serum *P* < 0.001; milk *P* < 0.001). The highest recorded prevalence of positive results for both serum and milk samples was 39% (Figure 1). No statistically significant difference (*P* = 0.668) was recorded in the prevalence of serum positive results, pre- and 72 h post-SICCT. Similarly no statistically significant difference (*P* = 0. 197) was recorded in *S*/*P* ratios of serum ELISA results pre- and 72 h post-SICCT. Both box plots and GEE analysis highlight an increase in both serum and milk *S*/*P* ratio readings subsequent to the 72 h sampling (Figures 2 and 3; Tables 2 and 3, respectively).

**Table 2:** **Multivariable GEE analysis of milk ELISA as a continuous (*S*/*P* % ELISA readings) and categorical (milk ELISA MAP positive/negative) dependent variable and independent variables**.

	**Continuous variable** (*S*/*P* % ELISA readings)			**Categorical variable** (Milk ELISA MAP positive/negative)			

**Dependent variable** Milk ELISA	**Coefficient**	***P* Value significant:** ***P* < 0.05**	**95% C.I.**	**Odds ratio**	***P* value**	**95% C.I.**	**Model** **(*P* value <0.001)**

**Independent variable**
Time point
June 20 vs. May^a^	17.2	**<0.001**	14.3, 20.2	11.1	**<0.001**	5.8, 21.0	
July 11 vs. May	5.43	**<0.001**	2.5, 8.4	2.7	**0.004**	1.4, 5.1	
July 24 vs. May	0.29	0.850	−2.7, 3.3	1.1	0.819	0.5, 2.3	
August 8 vs. May	0.94	0.537	−2.1, 3.9	1.1	0.831	0.5, 2.3	Sampling time point
August 21 vs. May	0.42	0.784	−2.6, 3.4	1.1	0.829	0.5, 2.3	Parity
Oct vs. May	1.51	0.322	−1.5, 4.5	1.8	0.105	0.9, 3.5	Breed
Nov vs. May	5.65	**<0.001**	2.6, 8.7	2.5	**0.007**	1.3, 4.9	Calving date
Parity
1^b^ vs. 2	−11.2	**<0.001**	−5.4, −17.0	0.3	**0.004**	1.6, 10.3	
2 vs. 3	11.2	**0.001**	17.5, 4.7	3.3	**0.025**	0.1, 0.9	
2 vs. 4	8.3	**0.002**	13.5, 3.1	2.5	**0.014**	0.2, 0.8	

*^a^May is the ELISA sample taken pre SICCT. ^b^Parity 1: 1^st^ lactation. No significant interactions identified with other independent variables. C.I., confidence interval. Coefficient, difference across the sample population. Statistically significant P values highlighted in bold*.

**Table 3:** **Multivariable GEE analysis of serum ELISA as a continuous (*S*/*P* % ELISA readings) and categorical (serum ELISA MAP positive/negative) dependent variable and independent variables**.

	**Continuous variable** (*S*/*P* % ELISA readings)			**Categorical variable** (Serum ELISA MAP positive/negative)			

**Dependent variable** Serum ELISA	**Coefficient**	***P* value significant:** ***P* < 0.05**	**95% C.I.**	**Odds ratio**	***P* value**	**95% C.I.**	**Model** **(*P* value <0.001)**

**Independent variable**
Time point
June 14 vs. May^a^	4.4	0.197	−2.3, 11.0	1.1	0.668	0.6, 2.1	
June 25 vs. May	33.8	**<0.001**	27.2, 40.5	10.7	**<0.001**	6.1, 18.8	
July 11 vs. May	37.9	**<0.001**	31.3, 44.6	6.4	**<0.001**	3.7, 11.1	
July 24 vs. May	17.0	**<0.001**	10.3, 23.7	2.3	**0.004**	1.3, 3.9	
August 8 vs. May	8.7	**0.010**	2.1, 15.4	1.3	0.392	0.7, 2.3	
August 21 vs. May	1.8	0.602	−4.9, 8.4	1.1	0.659	0.5, 1.8	
September 5 vs. May	4.0	0.241	−2.7, 10.6	1.0	0.998	0.5, 1.8	
October 1 vs. May	6.0	0.080	−0.7, 12.6	0.9	0.641	0.5, 1.6	Sampling time point
November 1 vs. May	11.1	**0.001**	4.5, 17.7	1.3	0.392	0.7, 2.3	Parity
Parity							Breed
1^b^ vs. 2	−29.4	**0.006**	−50.4, −8.4	0.4	0.053	0.2, 1.0	Calving date
3 vs. 2	−27.6	**0.015**	−50.0, −5.3	0.3	**0.018**	0.1, 0.8	
4 vs.2	−10.0	0.296	−28.8, 8.8	0.5	**0.047**	0.2, 1.0	
Calving Date							
February vs. January	−4.0	0.677	−22.9, 14.9	0.3	**0.003**	0.1, 0.6	
March vs. January	−24.0	0.066	−49.7, 1.6	0.2	**0.002**	0.1, 0.5	
April vs. January	−15.9	0.304	−46.3, 14.5	0.4	0.145	0.1, 1.4	
February vs. March	21.7	**0.036**	1.4, 41.9	1.7	0.241	0.7, 4.4	

*^a^May is the ELISA sample taken pre SICCT test*.

*^b^Parity 1 – 1st lactation. No significant interactions identified between independent variables. C.I., confidence Interval. Coefficient, difference across the sample population. Statistically significant P values highlighted in bold*.

Statistically significant differences between pre- and post-SICCT milk ELISAs were recorded until 43 days post-administration of PPD, examined as both a continuous and categorical variable (Table 2). The prevalence of ELISA serum positive samples was not statistically different from pre-SICCT levels by day 58, while serum ELISA *S*/*P* ratios remained significantly elevated for 71 days post-SICCT (Table 3). It should be noted that a significant elevation in *S*/*P* ratios post-SICCT was again noted in November (trial day 143) for both milk and serum samples (Tables 2 and 3). No significant second level interactions were identified between independent variables.

Spearman correlation analysis of matched serum and milk samples generated pre SICCT values of *r_s_* 0.73. Post SICCT values ranged from *r_s_* 0.55 to 0.79 with the highest levels recorded at post SICCT test 1 (*r_s_* 0.77) and post SICCT test 6 (*r_s_* 0.79).

Weekly fecal culture of consistently ELISA positive cows yielded negative results. A total of 10 animals yielded PCR positive results, 2 of which recorded positive results at each sampling time point. Veterinary examination did not yield any clinical signs of JD in these animals.

## Discussion

The Irish cattle population is subjected to a comprehensive and compulsory bTB eradication program, involving administration of the SICCT on at least an annual basis (15). The purpose of the current study was to investigate the impact of SICCT (i.e., administration of bPPD and aPPD) on both the within-herd prevalence of positive cows and ELISA *S*/*P* ratios in an Irish dairy herd. The results of the current study can provide useful guidance to farmers and veterinarians on the optimum period to conduct MAP ELISA testing in regions engaging in comprehensive testing for bTB using SICCT.

**Figure 4:**
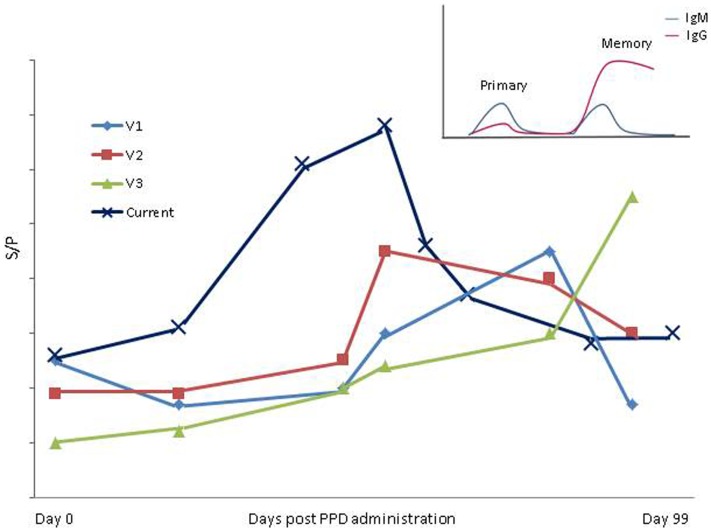
**Variation in period of influence of SICCT in present study compared to Varges et al. (21) is shown**. V1–V3; Approximate *S*/*P* results of positive cows identified using “in- house” ELISA by Varges et al. (21). Current; mean ELISA *S*/*P* results from entire herd in the present study. Insert; a schematic of primary and secondary/memory immune response [adapted from Tizard (25)].

Two international studies, one conducted in Brazil (21), and the second in the UK (24), have previously shown that tests for bTB interfere with MAP ELISA diagnostics. Varges et al. (21) reported ELISA interference occurring between 30 and 90 days post-PPD administration in bTB and MAP negative cattle. Of the 63 animals included in that study, 5 were classified as MAP ELISA positive post-PPD administration using both SICCT and single intradermal tuberculin test. Although the current study highlights a similar trend, the timescale over which interference is recorded differs. The increase in the number of animals detected ELISA positive post-SICCT and subsequent decrease to pre-SICCT prevalence occurred approximately 2 weeks earlier than the period of interference outlined by Varges et al. (21). The herd included in the current study had a history of recording serum MAP ELISA positive individuals (within-herd prevalence of 8%). This contrasts with the Brazilian study where cattle were confirmed MAP fecal culture negative prior to inclusion in the trial. It is possible, therefore, that cows used in the current study had been pre-sensitized to MAP or additional mycobacterial-related antigens. This being the case, it would be expected that a more rapid immune response would result, i.e., a secondary humoral memory response (25). The longer duration taken to record an IgG response and the lower proportion of ELISA positive cows identified post-PPD administration by Varges et al. (21) may be indicative of a slower primary immune response (Figure 4). As mentioned previously, Irish cattle are tested annually using SICCT from the age of 6-weeks, which may also account for the suggested memory response in Irish cattle in contrast to their Brazilian counterparts. Additionally, the studies differed in the ELISA kits used for MAP antibody detection and used limited sample populations. More extensive studies are, therefore, required to compare the performance of all commercially available MAP ELISA kits with regard to administration of both aPPD and bPPD and the need for development of more specific antigens to improve the specificity and sensitivity of currently available MAP ELISAs has been clearly highlighted. The inclusion of a greater number, and diversity, of animals and herds would also strengthen findings, as would continuation of a study over a number of years incorporating multiple TB tests.

Varges et al. (21) examined both the single intradermal and comparative bTB test. Interestingly, cross-reacting antibodies were detected using both SICCT and single intradermal test, while administration of aPPD alone did not elicit cross-reacting antibodies. This would suggest that bPPD may be responsible for generation of cross-reacting antibodies in the MAP ELISA kits examined in both studies. This is supported by a study by Olsen et al. (18), which highlighted reduced MAP ELISA specificity in animals experimentally infected with bTB. Interestingly, animals with natural bTB infection did not elicit cross-reacting antibodies (18), which may again suggest that the intradermal administration of bPPD, is indeed, the stimulant for generation of cross-reacting antibodies. Commercially available MAP ELISA kits incorporate an *M. pheli* absorption step to increase the specificity of the assay. Again, Olsen et al. (18) showed this to be an ineffective method of improving MAP ELISA specificity with regard to bTB. It may be that while pre-absorption with *M. pheli* is somewhat successful in reducing binding of *M. avium* antibodies, repeated administration of bPPD negates its effect in preventing non-specific binding. The potential for a cumulative effect of PPD administration (either avian or bovine) from multiple bTB tests over a number of years, therefore, requires thorough investigation to fully characterize the impact of SICCT on MAP ELISA testing.

In Ireland, herds restricted due to a positive bTB diagnosis (Directive 64/432/EEC), undergo two repeat tests at a 60-day interval. For herds operating under these restrictions, the results of the current study highlight that milk samples may be a more suitable test matrix than serum ELISA to avoid test interference. Similar to the results obtained by Lombard et al. (26), there was moderate agreement between serum and milk samples. Milk samples, however, took a shorter interval to return to pre-SICCT levels than serum in the current study. This may reflect the difference in IgG sub-classes between serum and milk and a lower milk IgG response (26). The post-SICCT period of elevated milk *S*/*P* ratios, however, may reflect a period of increased IgG production or IgG secretion from plasma to the mammary gland post-SICCT. This manifests as increased test sensitivity, stronger correlations highlighted between milk and serum results post-PPD administration. May et al. (24) also recorded significantly higher milk ELISA readings 4.5 weeks post-PPD administration in a UK herd. The limited statistical analysis completed by May et al. (24) and use of only a single testing timepoint post-SICCT presents difficulties in allowing direct comparisons between both datasets. Additionally, a number of regions in the UK administer the SICCT on one occasion every 4 years (27), a much longer testing interval than experienced by Irish herds. The differences highlighted between Varges et al., (21), May et al., (24) and the current study highlight the usefulness of examining the impact of SICCT on MAP ELISA results in multiple jurisdictions in order to more fully elucidate the impact of bTB testing on MAP diagnosis by ELISA.

It has previously been reported that exposure to environmental mycobacteria may yield low level protection against *M. tuberculosis* (28, 29). Hope et al. (30) also reported protection against *M. bovis* following exposure to *M. avium*, and that pre-exposure to *M. avium* results in an imprinting of memory against avian antigens onto T-lymphocytes. An amnestic response to environmental mycobacterial infection combined with continuous boosting of T cells in response to administration of PPD may, therefore, have the potential to assist in control of MAP at the animal level. In that regard, Ireland records a relatively low prevalence of MAP compared to additional milk exporting nations (31). For example, a total of 232 clinical cases of JD were reported in Ireland from 1995 to 2002 (32), yielding an average annual rate of approximately 0.0005%, given a cattle population of six million cattle (33). Additionally, Good et al. (34) reported that 20% of Irish herds contain at least one ELISA positive animal, again a relatively low prevalence (31). Given that environmental conditions in Ireland are conducive to the growth of mycobacteria (35), and that Irish farmers engage in high risk management practices with regard to spread of JD, e.g., widespread pooling of colostrum and milk for calf-feeding (36) (Kennedy et al. unpublished data), a higher prevalence of clinical cases and MAP ELISA positives might be expected. Another Irish study (37) recorded no significant associations between MAP seropositivity and milk production parameters, again contrasting with international studies (38, 39). It is our hypothesis that repeated annual administration of aPPD and bPPD in Ireland may induce a protective effect against MAP thereby lessening the clinical manifestations of MAP infection and resultant production losses. To more thoroughly investigate this hypothesis, it is necessary to complete in depth investigations as to whether the increase in antibody levels recorded post-PPD administration in the current study equates to an increased T-cell response, which would be required to achieve such a protective effect (40).

An advantage of the current study was the use of a compact spring-calving herd. This ensures that all cows examined were at a similar stage of lactation and physiological status. This allowed trends in MAP *S*/*P* % ratios over the latter half of lactation in a homogenous population to be examined. In agreement with a previous study (26), cows in late lactation were more likely to yield a MAP ELISA positive result using milk samples. The declining milk yields in late lactation result in a lessening of the dilution effect on antibody levels thereby increasing antibody concentrations (41). Interestingly, an increase in the prevalence of serum ELISA positives was also recorded in late lactation. This finding is in agreement with a Danish study (42). The increase in prevalence of serum ELISA positives in the current study corresponds with housing, which may increase the likelihood of exposure to mycobacterial antigens by increasing the potential for fecal contact. Nielsen et al. (42) also showed parity 2 and greater to be more likely to test ELISA positive relative to parity 1 cows, which is also highlighted in the current study. Parity 3 and 4 animals, however, were in general less likely to test positive than parity 2. The majority of Irish farmers target compact calving seasons (43) and strict culling practices are often in place (33). These culling practices may lead to less ELISA positive animals remaining in the system post second lactation. Results from this study indicate that age of animal at sampling and timing of JD ELISA tests relative to stage of lactation and time of bTB testing are important considerations when interpreting ELISA results.

## Conclusion

Administration of PPD as part of the bTB test corresponds to an increased prevalence of ELISA positives for JD. Diagnostic sampling for JD utilizing milk ELISA should be avoided in the 43-day period following the bTB test, with serum ELISA sampling not recommended for an additional 28 days. Based on the increase in antibody titers in MAP ELISA recorded post-PPD administration, it is our hypothesis that repeated annual administration of aPPD and bPPD may induce a protective effect helping to curtail the clinical manifestations of MAP infection.

## Conflict of Interest Statement

The authors declare that the research was conducted in the absence of any commercial or financial relationships that could be construed as a potential conflict of interest.
